# Lanreotide extended-release aqueous-gel formulation, injected by patient, partner or healthcare provider in patients with acromegaly in the United States: 1-year data from the SODA registry

**DOI:** 10.1007/s11102-012-0460-2

**Published:** 2013-01-13

**Authors:** Roberto Salvatori, Whitney W. Woodmansee, Mark Molitch, Murray B. Gordon, Kathleen G. Lomax

**Affiliations:** 1 Division of Endocrinology, Johns Hopkins University, 1830 East Monument Street #333, Baltimore, MD 21287 USA; 2 Division of Endocrinology, Diabetes and Hypertension, Brigham and Women’s Hospital/Harvard Medical School, Boston, MA USA; 3 Division of Endocrinology, Metabolism and Molecular Medicine, Northwestern University Feinberg School of Medicine, Chicago, IL USA; 4 Allegheny Neuroendocrinology Center, Division of Endocrinology, Allegheny General Hospital, Pittsburgh, PA USA; 5 Medical Affairs Endocrinology, Ipsen Biopharmaceuticals, Inc, Basking Ridge, NJ USA

**Keywords:** Acromegaly, Lanreotide depot, Extended-release, Observational study

## Abstract

Lanreotide depot (LD; commercial name Somatuline^®^ Depot) is an injectable, extended-release formulation of the synthetic somatostatin analog (SSA) lanreotide. In recent clinical trials, LD was found to be suitable for self or partner administration, avoiding the need to travel to a medical facility. The Somatuline^®^ Depot for Acromegaly (SODA) study is an ongoing, multicenter, observational study in the US investigating the efficacy, safety, convenience and symptom relief provided by LD in patients with acromegaly. Sub-analyses explore outcomes according to who administered the injection: patient, partner, healthcare provider (HCP) or a combination. Data reported here reflect one year of patient experience. Patients are eligible for inclusion if they have a diagnosis of acromegaly, are treated with LD and can give signed informed consent. Baseline data include patient demographics, previous acromegaly treatment and investigations, GH and IGF-I levels, LD dose and dose adjustment frequency. Symptom frequency, injection pain and treatment convenience are assessed using patient-reported questionnaires. As of 18 April 2012, 166 patients had enrolled in SODA. Most (72 %) achieved normal IGF-I levels after 12 months of LD treatment. Disease control was similar in self or partner injectors and in patients who received injections from their HCP, although self or partner injecting was deemed more convenient. LD was well-tolerated irrespective of who performed the injection. Self injection led to more injection-site reactions, but this did not increase the rate of treatment interruption. Acromegaly symptoms remained stable. Biochemical, safety and convenience data support the clinical validity of injecting LD at home.

## Introduction

Acromegaly is a rare disorder that results from excessive production of growth hormone (GH), usually due to a benign pituitary adenoma. Affecting approximately 40–125 people per million in the United States (US) [1], and possibly more [2–4], acromegaly is characterized by symptoms related to multiple body systems and increased risk of all-cause mortality [5]. Treatment typically entails surgical excision of the pituitary adenoma to normalize GH secretion, and to relieve compression symptoms in cases of larger tumor mass. However, complete tumor removal may not be possible if, as often happens, adenomas are large at the time of diagnosis, or in occasional cases when surgery is contraindicated or declined. As a result, over 50 % of patients have active residual disease, defined by increased levels of GH, and/or its physiological mediator, insulin-like growth factor-1 (IGF-I), leading to persistent clinical symptoms, impaired health-related quality of life and increased mortality [6–8]. A recent systematic review of the literature determined that mortality, morbidity and cost are all higher in patients with biochemically uncontrolled acromegaly than in those with GH levels <2.5 μg/L and IGF-I normal for age and gender [9]. As such, patients with uncontrolled disease typically receive pharmacological therapy such as somatostatin analogs (SSAs), the GH receptor antagonist pegvisomant or, less commonly, dopamine agonists or radiotherapy, to further reduce the symptoms and long-term consequences of unregulated GH secretion [1, 10].

Lanreotide depot (LD) is a synthetic octapeptide SSA that binds to somatostatin receptors Type 2 and—to a lesser degree—Type 5, inhibiting GH secretion and reducing IGF-I levels [11]. The long-acting, extended-release aqueous-gel formulation lanreotide depot is known in the US as Somatuline^®^ Depot [12], and as Somatuline Autogel^®^ in the rest of the world. LD is approved in the US for the long-term treatment of acromegaly in patients with inadequate response, or contraindications, to surgery or radiotherapy [12]. LD is supplied as a pre-filled syringe for deep subcutaneous injection of 60, 90 or 120 mg, does not require reconstitution, and was recently demonstrated in a 6-month clinical trial to be suitable for self or partner administration thus avoiding travel to a medical facility [13]. Whether LD may reliably be self-administered in the real-world setting remains unknown.

The Somatuline^®^ Depot for Acromegaly (SODA) study is an ongoing, multicenter, observational study in the US, investigating the efficacy, safety, treatment convenience and symptom relief provided by LD in patients with acromegaly. Several post hoc sub-analyses have been conducted, including differences in outcome based on who administered the injection: patient, partner, healthcare provider (HCP) or a combination of injectors. The data reported in this manuscript reflect 1 year of patient experience in this ongoing study.

## Patients and methods

### Patients

Patients are eligible for inclusion in the SODA study if they have a clinical diagnosis of acromegaly, are treated with LD (including patients for whom LD is newly prescribed and those switched from other agents) and are competent to give signed informed consent. Those with symptomatic, untreated gallstones or known sensitivity to SSAs are ineligible. There is no limit on time from prior surgery or radiation therapy and patients can be enrolled at any time after starting the drug. Patients who have never received any form of octreotide and those who start LD within 30 days prior to enrollment are considered treatment-naïve. All eligible patients are included in the study and continue to receive LD as prescribed by their physician for the duration of their participation. The day on which the consent form is signed is considered the enrollment date.

### Study design and assessments

The SODA study is carried out in academic and private treatment centers in the US. Baseline characteristics include patient demographics, previous acromegaly treatment and investigations, and hormone levels (GH and IGF-I). LD dose and frequency of dose adjustment are recorded at every study visit after enrollment. As this is a non-interventional, observational study, the frequency of study visits, biochemical testing, radiological, echographic and sonographic evaluation is determined by the treating physician, and thus not all data points are available for all patients. Efficacy is assessed using serum IGF-I and GH concentrations evaluated at either a central or local laboratory with the recommended time points being at 3 and 6 months, then yearly. Investigators record whether the levels are normal, elevated or low for their particular hormone assay. Additional secondary analyses include safety, symptom burden and treatment convenience. Safety is evaluated by physical examination and recording of adverse events (AEs), which are categorized in the protocol as targeted (known to be associated with LD and other SSAs, such as injection site reactions, bradycardia, diarrhea, or cholelithiasis) or unexpected. Symptom burden (frequency), injection pain and treatment convenience are assessed by administering the two patient-reported questionnaires to each patient. The symptom questionnaire asks patients whether they experience specific symptoms always/most of the time/sometimes/rarely/never; it is recommended to be administered at enrollment, 6, 12 and 24 months after enrollment, and every subsequent 6 months until study completion, or at interval visits if these occur outside of the planned schedule. The convenience questionnaire inquires about who administers SSA injections, how long injections take to administer (including travel time to reach the clinic, if necessary) and whether injections are convenient, painful and/or technically difficult. It follows the same schedule as the symptom questionnaire except that the first post-enrollment questionnaire is administered at 12 months. Questionnaires are available in “Appendix”.

For this report, biochemical control (GH and IGF-I levels) was analyzed using the 1-year completer population, defined as all patients with IGF-I levels available at enrollment and month 12 (n = 87). LD dose, symptom control, pain and convenience were analyzed for all patients who had the respective data available at enrollment and after one year; as such, the observed data reflect different patient numbers in each group. Safety analyses were conducted on all enrolled patients (n = 166). Data were categorized according to who administered the LD injections: patient, partner, HCP or any combination of patient, partner and HCP (the ‘Combination/other’ injector group). Statistical analyses were primarily descriptive. Data reported in this paper reflect a data cut on 18 April 2012.

## Results

### Patients

As of 18 April 2012, 166 patients had enrolled in SODA, 104 (63 %) from academic medical centers, and 62 (37 %) from private practice sites, across 22 states. Baseline characteristics of the enrolled population are presented in Table 1. Acromegaly was caused by a pituitary adenoma in almost all patients (98 %). The majority (80 %) had undergone pituitary surgery, 20 % had received radiation therapy and 123 subjects (74 %) had received previous pharmacological treatment in the form of SSAs, pegvisomant or a dopamine agonist; 19 (11 %) had not received any previous acromegaly treatment or had started LD ≤30 days prior to enrollment (collectively considered treatment-naïve for this analysis).

**Table 1:** Baseline characteristics of 166 patients at time of enrollment in SODA Study

	Injection administered by				
	Patient always (n = 26)	Partner always (n = 41)	HCP always (n = 58)	Combination/other (n = 41)	All patients (n = 166)
Gender, % M/F	35/65	59/42	43/57	66/34	51/49
Mean age, years (range)	52 ± 12 (23–73)	52 ± 16 (22–84)	49 ± 17 (13–86)	49 ± 13 (25–73)	50 ± 15 (13–86)
Time since diagnosis, months (mean)	99 ± 77	65 ± 63	98 ± 117	96 ± 105	89 ± 98
Etiology of acromegaly, n (%)^a^					
Pituitary adenoma	26 (100)	41 (100)	56 (97)	40 (98)	163 (98)
McCune-Albright syndrome	0	1 (2)	1 (2)	0	2 (1)
Other^b^	0	0	1 (2)	1 (2)	2 (1)
IGF-I level measured^c^ (n)	25	36	52	40	153
High, n (%)	10 (40)	15 (42)	28 (54)	23 (58)	76 (50)
Normal, n (%)	14 (56)	21 (58)	23 (44)	16 (40)	74 (48)
Low, n (%)	1 (4)	0	1 (2)	1 (3)	3 (2)
Unknown, n (%)	1 (4)	5 (12)	6 (15)	1 (3)	13 (8)
Peak glucose-suppression GH level (n)	6	4	6	8	24
Median, ng/mL	1.1	0.7	2.5	2.0	1.8
Trough GH ≤2.5 ng/mL, n (%)	5 (83)	3 (75)	3 (50)	5 (63)	16 (67)
Trough GH ≤1 ng/mL, n (%)	3 (50)	3 (75)	2 (33)	3 (38)	11 (46)
Prior pituitary surgery, n (%)	24 (92)	29 (71)	44 (76)	36 (88)	133 (80)
Prior radiation therapy, n (%)	2 (8)	5 (12)	15 (26)	12 (29)	34 (20)
Prior medical therapy, n (%)^d,e^	20 (77)	28 (68)	45 (78)	30 (73)	123 (74)
SSA	15 (58)	19 (46)	39 (67)	24 (59)	97 (58)
Short-acting octreotide	0	1 (2)	12 (21)	2 (5)	15 (9)
Long-acting octreotide	15 (58)	18 (44)	27 (47)	22 (54)	82 (49)
Dopamine agonist	9 (35)	17 (42)	14 (24)	11 (27)	51 (31)
Pegvisomant	5 (19)	2 (5)	12 (21)	9 (22)	28 (17)
None (treatment-naïve)	2 (8)	7 (17)	5 (9)	5 (12)	19 (11)

Given the non-interventional nature of the study, not all datapoints are available for all patients

*GH* growth hormone, *GHRH* growth-hormone releasing hormone, *IGF-I* insulin-like growth factor-1, *SSA* somatostatin analog

^a^Categories are not mutually exclusive; two subjects were categorized as having a GH-secreting macroadenoma and a pituitary adenoma; one subject had a pituitary adenoma and McCune-Albright syndrome

^b^Pituitary enlargement with high IGF-I, suggesting a probable GHRH-secreting pinealoma

^c^The proportion of IGF-I samples analyzed centrally was 20 % at enrollment and 27 % at 12 months; the remaining samples were analyzed in local institutional laboratories. Values determined to be high, normal or low by investigator

^d^Values based on case report forms which did not identify patients using lanreotide depot

^e^Therapies were not mutually exclusive

IGF-I levels were elevated in half of the population (76/153) at enrollment and 28 % (24/87) of 1-year completers. The proportion of samples analyzed at local laboratories was 80 % at enrollment and 73 % at 12 months; the remaining samples were analyzed at a central laboratory. The majority of patients were taking 90 mg LD (Table 2). Almost all enrolled patients (95 %) received LD injections every 28 days and one was receiving 120 mg at the extended dosing interval of every 42 days or longer. More patients received injections from their HCP (58/166; 35 %) than from other sources; 16 % self-injected, and the remaining 50 % were evenly split between those who had injections administered by their partner and those who used a combination of methods.

**Table 2:** Lanreotide depot dose (n, %) at enrollment and after 12 months of treatment

	Patient always	Partner always	HCP always	Combination/other	
Dose at enrollment (mg)	n = 26	n = 41	n = 58	n = 41	All patients (n = 166)
60	1 (4)	6 (15)	11 (19)	9 (22)	27 (16)
90	18 (69)	24 (59)	29 (50)	23 (56)	94 (57)
120	7 (27)	11 (27)	18 (31)	9 (22)	45 (27)

Dose at 12 months (mg)	n = 23	n = 38	n = 50	n = 35	All patients (n = 146)
60	1 (4)	6 (16)	7 (14)	7 (20)	21 (14)
90	10 (44)	19 (50)	21 (42)	12 (34)	62 (43)
120	12(53)	13 (34)	22 (44)	15 (43)	62 (43)

Thirty-three patients discontinued the study during the first year, six due to personal choice, three for financial reasons, two died (one congestive heart failure and one cardiac arrest) and another 14 discontinued for other reasons (including pregnancy, change of physician, normal lab tests or breach of protocol). Eight were lost to follow-up. Differences in discontinuation rates between injector subgroups were not significant.

### Biochemical control: IGF-I and GH levels

**Fig. 1:**
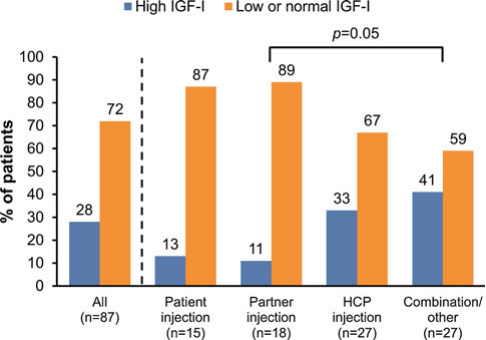
IGF-I levels after 12 months’ treatment with lanreotide depot (n = 87). The ‘Combination/other’ group comprises patients who received injections from any combination of injectors [self, partner, (HCP)]

In the 1-year completer population (n = 87), the majority (63/87; 72 %) demonstrated biochemical control after 12 months, as evidenced by IGF-I levels below the upper limit of normal (Fig. 1); there was no significant difference between previously treated and treatment-naïve patients. IGF-I levels were normalized in a similar percentage of self (13/15; 87 %) and partner injectors (16/18; 89 %) and a somewhat lower proportion of subjects who received injections from their HCP (18/27; 67 %) or a combination of injectors (16/27; 59 %) (*p* = 0.05 between partner-injectors and the combination/other group; all other pairwise comparisons were non-significant) (Fig. 1). However, when both groups of ‘home injectors’ (self and partner injectors) were compared with both groups of ‘office injectors’ (HCP and combination/other groups), a significantly greater proportion of home injectors had normalized IGF-I levels after 12 months [29/33 (88 %) vs. 34/54 (63 %); *p* = 0.01 on Fisher’s Exact test]. Mean fasting GH level after 12 months, based on data available in 50 patients, was 1.7 ± 2.2 ng/mL; levels were ≤ 2.5 ng/mL in 40/50 (80 %) patients and <1 ng/mL in 30/50 (60 %) patients.

### Lanreotide depot dosing

LD dose information was available for 146 patients at 12 months (Table 2). In total, 42 (29 %) had dose adjustments in the first year of enrollment in the study. The proportion of subjects taking a 90 mg dose decreased in the first year while the proportion taking 120 mg increased, representing a general trend towards dose increase. Dose adjustment was most commonly prompted by elevated IGF-I levels (24/42; 57 %; *p* = NS between groups); other reasons included low IGF-I levels (9/42; 21 %), high GH levels (7/42; 17 %) and other reasons (10/42; 24 %). As at enrollment, the majority (89 %) were administering injections every 28 days after 12 months of treatment.

### Symptoms and convenience

A total of 100 patients (100/166; 60 %) completed the symptom questionnaire both at enrollment and 12 months. At enrollment, tiredness was the most common symptom (72/100; 72 %) followed by snoring (68/100; 68 %), pain (64/100; 64 %), sweating (58/100; 58 %), and headache (32/100; 32 %). After 12 months, all symptoms remained stable in these 100 patients.

**Fig. 2:**
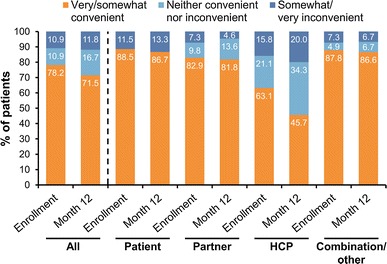
Self-reported convenience of lanreotide depot according to who injected the treatment. The ‘Combination/other’ group comprises patients who received injections from any combination of injectors [self, partner, healthcare provider (HCP)]

There was a second questionnaire concerning pain and convenience filled out by the patients. At enrollment, 33 % (54/166) of the patients reported LD injections to be painless, 55 % (92/166) described them as mildly/moderately painful and 11 % (19/166) found injections very/extremely painful. Responses after 12 months of treatment with LD were similar to those at enrollment. Convenience data (Fig. 2) indicated that overall, for the 102 patients who completed this questionnaire at both enrollment and month 12, 72 % (73/102) of patients found LD very or somewhat convenient, although HCP injection was considered less convenient than self or partner injection at enrollment and at 12 months.

### Adverse events

In all, 61 % (101/166) of patients reported at least one AE, with minimal difference between injector subgroups. The most common targeted AEs (occurring in ≥10 % of patients) were arthralgia, headache and gastrointestinal disturbances (Table 3). Injection site reactions were considerably more common when patients injected themselves than when they were injected by someone else [19 % (5/26) self injectors versus 2 % (1/41) partner injectors; *p* < 0.05]. Eleven percent of patients (18/166) reported a total of 41 serious AEs (SAEs). Cerebrovascular accident (n = 3) was the most common SAE, followed by pneumonia, acute renal failure, and urinary tract infection (n = 2 for each). In three patients, SAEs were considered possibly related to LD treatment (depression/suicidal ideation, atrial fibrillation, cluster headache). Both deaths were considered unrelated to treatment by the investigator.

**Table 3:** Targeted AEs reported by ≥10 % of all patients

	Injection administered by				
	Patient (n = 26)	Partner (n = 41)	HCP (n = 58)	Combination/other (n = 41)	All patients (n = 166)
Number of patients with ≥1 targeted AE (n, %)	15 (58)	23 (56)	30 (52)	23 (56)	91 (55)
Targeted AEs					
Arthralgia	4 (15)	9 (22)	17 (29)	6 (15)	36 (22)
Headache	8 (31)	10 (25)	9 (16)	9 (22)	36 (22)
Diarrhea	1 (4)	12 (29)	8 (14)	11 (27)	32 (19)
Abdominal pain	2 (8)	10 (24)	10 (17)	9 (22)	31 (19)
Nausea	0	10 (24)	9 (16)	5 (12)	24 (15)
Constipation	3 (12)	4 (10)	8 (14)	7 (17)	22 (13)
Flatulence	2 (8)	7 (17)	4 (7)	8 (20)	21 (13)
Injection site reaction	5 (19)	1 (2)	4 (7)	3 (7)	13 (8)

*AEs* adverse events, *HCP* healthcare provider

## Discussion

In this real-world, observational study in patients with active acromegaly treated with LD, alone or as combination therapy, most (72 %) patients achieved normal IGF-I levels after 1 year, indicating attainment of biochemical disease control. Approximately one-third of patients required LD dose adjustment during the year, typically an increase from 90 to 120 mg due to persistently elevated IGF-I levels. The percentage of patients achieving biochemical control in this study is higher than has generally been reported previously with SSAs [14], and likely reflects an enrollment bias since subjects who were poorly or non-responsive to LD are less likely to be enrolled and/or to continue receiving LD in this real-life study. Our data were too few to allow comparison of response rates between treatment-naïve and previously treated subjects. More importantly, disease control was similar in those who self or partner injected when compared to patients who always received injections from their HCP. Furthermore, comparison of exclusively home injectors (patient or partner only) with patients who received office injections revealed that a significantly greater proportion of exclusively home injectors achieved biochemical control after 1 year of LD treatment, which may reflect a tendency of patients who respond better to visit the physician’s office less often. In conjunction with stable symptoms, good tolerability, and data indicating that self and partner injecting were more convenient than injections administered by a HCP, these data support the clinical validity and lifestyle benefits of injecting LD at home. The only AE that occurred with greater frequency among self injectors than other groups was injection site reactions, which were not associated with a higher rate of withdrawal from the study.

Finding an alternative to visiting the HCP’s office may be particularly important for patients who are busy, limited in their mobility, or unable to easily access a local clinic for injections. In addition to improving convenience, injection at home may reduce direct (transportation, parking) and indirect (time away from work; time spent by HCPs administering treatment) costs to the patient and their healthcare system. Data from other therapy areas in which treatment has traditionally been administered by HCPs suggest that injection at home can lead to similar or superior treatment adherence and quality of life compared with HCP injections when injectors are competent in the required techniques [15–18]. Such findings lend weight to strategies that move beyond the typical reliance on HCP-only injections to include partners and patients themselves.

The validity of self/partner injection of LD has been demonstrated in two non-randomized, open-label controlled studies in acromegaly patients, one in the US (n = 59) [13] and one in the UK (n = 30, 15 receiving home injections) [19], both of which showed that injections could be correctly administered by self/partner without compromising efficacy or safety in most patients. In the UK study, 14/15 (93 %) patients who elected to receive home injections were able to successfully inject LD without supervision, as determined by HCP confirmation of accurate injection technique and maintenance of biochemical (IGF-I and GH) control for the 40-week duration of the study. In the US study, 33/59 (56 %) patients switched from octreotide LAR (injected by an HCP) to self- or partner-injected LD [13]. According to their HCPs, all 41 patients and 18 partners correctly administered LD injections by week four. While the UK and US studies recruited patients treated primarily at academic centers who were amenable to enrolling in a clinical trial, the SODA study includes a more heterogeneous patient population treated at both private and academic centers. Our findings therefore corroborate and extend the aforementioned study data, providing support for the validity of self or partner injecting in the real-world clinical environment.

The SODA study has a number of limitations. Firstly, it is an observational study and not a randomized controlled trial. As such, the causative role of LD on the reported outcomes is hard to determine or quantify. Secondly, an enrollment bias toward SSA-responsive patients is evident. Thirdly, the SODA population is highly heterogeneous due to enrollment at different stages of disease and treatment. Therefore, while the results provide a real-life snapshot of the SODA patient population at 1 year, the conclusions that can be drawn with respect to particular patient groups are limited. Finally, selection bias among treating physicians toward prescribing this medication for patients who are more likely to successfully self-administer the drug cannot be excluded. Such limitations notwithstanding, these 1-year data suggest that administration of LD at home by self or partner provides similar biochemical control to injections administered in the healthcare provider’s office, with similar tolerability and greater convenience. Longer-term data and/or data from controlled trials will be necessary to corroborate these findings.
